# Simulated Patient Role-Plays with Consumers with Lived Experience of Mental Illness Post-Mental Health First Aid Training: Interrater and Test Re-Test Reliability of an Observed Behavioral Assessment Rubric

**DOI:** 10.3390/pharmacy9010028

**Published:** 2021-01-24

**Authors:** Sarira El-Den, Rebekah J. Moles, Randi Zhang, Claire L. O’Reilly

**Affiliations:** Faculty of Medicine and Health, School of Pharmacy, The University of Sydney, Sydney, NSW 2006, Australia; rebekah.moles@sydney.edu.au (R.J.M.); rzha8341@uni.sydney.edu.au (R.Z.); claire.oreilly@sydney.edu.au (C.L.O.)

**Keywords:** reliability, mental health education, assessment, simulated patient, observed behavioral measurement

## Abstract

Mental Health First Aid (MHFA) training teaches participants how to assist people experiencing mental health problems and crises. Observed behavioral assessments, post-training, are lacking, and the literature largely focuses on self-reported measurement of behaviors and confidence. This study explores the reliability of an observed behavioral assessment rubric used to assess pharmacy students during simulated patient (SP) role-play assessments with mental health consumers. Post-MHFA training, pharmacy students (*n* = 528) participated in SP role-play assessments (*n* = 96) of six mental health cases enacted by consumers with lived experience of mental illness. Each assessment was marked by the tutor, participating student, and consumer (three raters). Non-parametric tests were used to compare raters’ means scores and pass/fail categories. Interrater reliability analyses were conducted for overall scores, as well as pass/fail categories using intra-class correlation coefficient (ICC) and Fleiss’ Kappa, respectively. Test re-test reliability analyses were conducted using Pearson’s correlation. For interrater reliability analyses, the intra-class correlation coefficient varied from poor-to-good to moderate-to-excellent for individual cases but was moderate-to-excellent for combined cases (0.70; CI 0.58–0.80). Fleiss’ Kappa varied across cases but was fair-to-good for combined cases (0.57, *p* < 0.001). For test re-test reliability analyses, Pearson’s correlation was strong for individual and combined cases (0.87; *p* < 0.001). Recommended modifications to the rubric, including the addition of barrier items, scoring guides, and specific examples, as well as the creation of new case-specific rubric versions, may improve reliability. The rubric can be used to facilitate the measurement of actual, observed behaviors post-MHFA training in pharmacy and other health care curricula.

## 1. Introduction

Mental and addictive disorders affect over one billion people, globally [1]. Furthermore, suicide is currently among the leading causes of death, worldwide [2]. Given the high prevalence of mental illness and the lack of help sought by those affected [3,4], the role of primary healthcare professionals, such as pharmacists, in providing initial care has been recognized [5,6]. Research has, however, indicated that health professionals may lack adequate training and education in their university curricula to enable them to confidently care for people at risk of suicide [7]. Hence, additional training may be required to equip health professionals with these skills. One such training program available internationally and increasingly embedded into university curricula [8] is Mental Health First Aid (MHFA), which teaches participants how to assess and assist those experiencing mental health problems and crises, including suicide [9].

Participants who have completed MHFA training have been taught how to apply the MHFA Action Plan, through various activities, including role-plays and case studies [9]. Hence, in theory, a participant who has completed MHFA training should be able to provide appropriate first aid to someone experiencing a mental health problem or crisis, such as suicide. Evaluations of MHFA training among a diverse range of participant populations indicate that it leads to self-reported improvements in accurately identifying mental illness and providing help to consumers experiencing mental health problems and crises [10,11]. The impact of MHFA training on students has also been explored, and it has been shown to lead to improvements in knowledge, attitudes, and self-reported confidence in providing care [12,13]. Furthermore, self-reported measures of post-training intentions and behaviors have been developed and shown to have sound test re-test and interrater reliability [14].

However, there is a lack of literature exploring observed behavioral assessments post-MHFA training [8]. This is important to explore as self-reported behaviors may not necessarily translate to improvements in participants’ actual behaviors. The tendency to over- or underestimate actual behaviors in self-report measures is common, and there is evidence supporting this phenomenon in relation to the measurement of behaviors, such as hand hygiene behavior [15] and physical activity [16]. Furthermore, in the pharmacy education literature, among students who completed online asthma first-aid training, only 29% were able to effectively demonstrate life-saving skills post-training [17]. Moreover, self-reported measures are prone to various forms of response bias, including social desirability bias [18]. Observed behavioral measures are integral forms of educational assessment, and it is important that they are developed and evaluated in a transparent manner to ensure standardized assessment across settings [18].

MHFA has been implemented into the final year undergraduate Bachelor of Pharmacy (BPharm) curriculum at Sydney Pharmacy School, since 2015. Post-MHFA training, pharmacy students must participate in or observe a simulated patient role-play with a consumer with lived experience of mental illness who is enacting a mental health problem or crisis, such as suicide, as part of their assessments within a Unit of Study. After the role-play, the student receives on-the-spot performance feedback in a safe learning environment from the tutor, consumer, and their peers. They also complete a self-assessment, allowing them to reflect on their learning during the role-play. These assessments were developed to support students’ learning and to enable them to practice and ultimately demonstrate the desired outcome [19], namely, providing appropriate first aid to a person experiencing a mental health problem or crisis. Research exploring the impact of the simulated patient role-play assessments has shown that they are more effective in improving self-reported competence and confidence than MHFA training alone, regardless of whether the student directly participated in or observed the role-play [20]. Furthermore, preliminary evidence indicates that participants may over-estimate or under-estimate their abilities in self-reported evaluations of confidence, when compared to their performance during simulated patient role-plays [21]. Therefore, despite improvements in self-reported confidence post-training, self-reported confidence assessments alone may not provide an accurate measure of skill acquisition and application, and objective observational measures are required to explore how MHFAiders apply their skills and whether they do so in a manner consistent with the content taught in MHFA training. Nonetheless, self-reported measures continue to be valuable as they can promote learning by providing opportunities for participants to reflect on their learning and articulate, in their own words, the behaviors they want to change [22,23,24].

Self-reported measures of confidence to provide MHFA are available and often comprise item stems with Likert-scale response options [10,21]; however, when assessing observed behaviors, a rubric is needed to aid assessors in determining whether each key action has been completed. Rubrics are measurement instruments that are often used to measure participants’ skills post-training [25]. A well-designed, reliable rubric can facilitate teaching [25] and ensures consistent measurement across time and assessors [26]. It can also highlight where students may perform poorly, and thereby, indicate that further support is needed within the curriculum [27].

The simulated patient role-plays are assessed using a 12-item rubric developed by the research team, comprised of accredited MHFA instructors, MHFAiders, mental health and education researchers, and pharmacists, based on the MHFA Action Plan, ALGEE [9] (Approach the person, assess and assist with any crisis; **L**isten and communicate non-judgmentally; **G**ive support and information; **E**ncourage the person to get appropriate professional help; **E**ncourage other supports) and a scoring system developed by MHFA researchers [28], lending evidence to its content validity. However, the rubric’s reliability as an assessment tool across time and markers has not been explored. Due to the importance of developing reliable rubrics to ensure that assessors are marking students in a reliable manner [26], this study aimed to investigate the reliability of the rubric designed to measure observed MHFA skills during simulated patient role-play assessments. More specifically, the objectives of this study were to:

1. Explore the interrater reliability of the rubric;
2. Explore the test re-test reliability of the rubric;
3. Recommend modifications to the rubric based on the reliability analyses.

## 2. Materials and Methods

In the Professional Practice Unit of Study, in the final year of the BPharm degree, all students are required to demonstrate their first aid skills across various domains, including MHFA, anaphylaxis, asthma, and angina. Students are randomly allocated to different domains and are unaware of their allocation when they attend their assessment tutorial. Each of these assessment tasks are typically enacted by one student in front of the rest of the class of up to 10 students. Students allocated to the MHFA assessment are required to participate in a simulated patient role-play with a consumer with lived experience of mental illness, while a tutor assesses their performance. These assessments have been evaluated and found to be valuable to both students and consumers [29].

Ten mental health consumer educators (consumers with lived experience of mental illness) from One Door Mental Health [30] in New South Wales were employed from 2016 to 2018 to participate in the role-plays. Six MHFA scenarios (Appendix A) were developed, based on DSM-V Diagnostic Criteria [31], between 2016–2018, for this purpose. Each year, two new scenarios with similar characteristics were developed, in that they involved consumers presenting to the pharmacy with direct prescription or over-the-counter product or symptom-based requests, due to symptoms of depression and/or anxiety. Two cases involved a consumer who had no suicidal thoughts, two cases involved a consumer who had suicidal thoughts, but had not considered a plan for suicide, and two cases involved a consumer who had suicidal thoughts and had considered a plan for suicide. Depression and anxiety symptoms were used as they represent the two most common mental illnesses in Australia [4]. Suicidal thoughts and behaviors were incorporated into the scenarios as previous research has demonstrated that students often struggle to assess for suicide directly [21], and suicide education is lacking from health care curricula [7]; hence, assessments were integrated into the curriculum to provide students with an opportunity to practice assessing for suicide, post-MHFA training.

The role-plays were audio-recorded in 2017 and 2018 with student consent (audio-recordings were not available for 2016), to allow for quality assurance of assessments in the curriculum, as well as quantitative and qualitative analyses of student performance, including test re-test reliability analyses. An announcement was made through the Unit’s Learning Management System site, informing students of the opportunity to participate in the research over the semester. Upon entering the classroom, the tutor provided students with the Participant Information Statement and the consent form, if they agreed to participate.

The enacted case was assessed separately by the tutor during and after the role-play and then by the consumer and student (self-assessment) immediately after the role-play. After assessing performance using the rubric, on-the-spot performance feedback discussions, and debrief between the tutor, mental health consumer educator, participating student, and observing students occurred.

This study was approved by The University of Sydney Human Research Ethics Committee (Project Number: 2015/626).

### 2.1. Rubric

The original rubric consisted of 12 items, with each item scored from 0 to 2 (0 points = incorrect, inappropriate, or missing behaviors; 1 point = partial demonstration of appropriate behaviors; 2 points = full demonstration of appropriate behaviors). The rubric also contained example phrases and actions for items 4, 6, 7, 9, 10, and 12 (Table 1). It was intended to be used universally to assess MHFA participants’ observed behaviors during simulated patient role-play assessments across various mental illnesses and crises, such as depression, suicidal thoughts and behaviors, and anxiety. It has been used in previous studies to explore pharmacy students’ performance during simulated patient role-plays [20,21]. Pass/Fail categories can be derived from the rubric. A “Pass” mark is given when a student’s overall score is 12 or greater, and the student assesses for suicide (item 5). If the simulated patient was experiencing suicidal thoughts and behaviors, then the student is required to perform the appropriate actions (item 9), as well, to pass. A “Fail” mark is given when a student’s overall score is less than 12 and/or the student does not assess for suicide (item 5). Even if a student receives a score greater than 12 and assesses for suicide, if he/she does not perform the appropriate actions (item 9) for a simulated patient experiencing suicidal thoughts and behaviors, then the student would fail the assessment.

### 2.2. Data Analysis

Interrater reliability (IRR) and test-retest reliability analyses were conducted using IBM SPSS Statistics Version 24 [32]. Each of the six cases was analyzed individually, and data from all cases was also combined and analyzed. Any missing data or data collected without students’ consent (as they were being marked for the unit of study they were enrolled in, regardless) were omitted from the analysis. Overall mean scores and pass/fail rates for each marker were also calculated. Non-parametric tests, namely, the Wilcoxan Signed Rank Test and McNemar’s Test, were used to determine significant differences across mean scores and pass/fail categories, across raters, respectively.

#### 2.2.1. Interrater Reliability Analyses

The tutor acted as an observer who only marked the role-play, but did not participate in the role-play. Marking by the consumer and student was not conducted during the interaction, to ensure that the consumer and student focused on the role-play interaction only. Rather, the consumer and student were given the rubric to mark after the conclusion of the simulated patient role-play assessment when the audio-recording was switched off. Data collected from the three raters between 2016 and 2018 were used for IRR analyses. Three types of IRR analyses were conducted, guided by published evidence exploring the interrater reliability of audio-recordings [33]:

1. Overall percentage agreement of markers, for each item across all combined cases.
2. Intra-class Correlation Coefficient (ICC) test using one-way random effects model, absolute agreement, multiple raters, to analyze the IRR of overall scores (i.e., marks out of 24) of the three markers (tutor, consumer, and student),
3. Fleiss’ Kappa was used to analyze the IRR of pass/fail categories across the three markers, as it is an appropriate measure of IRR for categorical data. Fleiss’ Kappa was also appropriate as this study involved more than two markers, and because this statistic “does not assume that the same raters have assessed all items” [34], as in the current study, different tutors, consumers, and students participated in the interactions, but each role-play was marked by one tutor, one consumer, and one student.

#### 2.2.2. Test Re-Test Reliability Analyses

Test re-test reliability analyses were conducted using available audio-recordings from the 2017 and 2018 cohorts. One researcher (R.Z.), who was not present during or involved in the live assessments, listened to and independently marked the student based on the audio recordings of the simulated patient role-plays at two separate time points, four weeks apart (December 2018 and January 2019). Item number 11 (*Good non-verbal communication*) could not be assessed using audio recordings and was omitted from the test re-test reliability analyses. Hence, the overall score for this analysis was 22, rather than 24. Test re-test analyses were conducted using Pearson’s correlation (*p* < 0.05).

## 3. Results

Between 2016 and 2018, 528 BPharm students completed MHFA training as part of their core curriculum. Due to the large number of students enrolled, multiple MHFA training sessions occurred across the semester. Approximately 1–10 weeks post-training, 102 simulated-patient role-plays assessing MHFA skills were conducted, of which 96 were considered eligible for inclusion in this study as students had provided consent for participation (94.1% consent rate). Students were randomly allocated to one of six MHFA cases. Table 2 illustrates the overall percentage agreement among markers for each item, across combined cases. Item 12 (*“Appropriate follow-up actions”*) had the lowest percentage agreement at 39.93%, while item 5 (*“Asks if the patient is having suicidal thoughts”*) had the highest percentage agreement at 94.45%.

Each student was marked by three markers, resulting in 288 rubrics and associated scores to be used in the IRR analyses. The mean scores were 17.15, 19, and 17.79 out of 24, as marked by tutors, consumers, and students (self-assessment), respectively. Using Wilcoxon Signed Ranks Tests, significant differences were identified between consumer and student scores (*p* = 0.002) and consumer and tutor scores (*p* < 0.001), but not tutor and student scores (*p* = 0.2). The ICCs for overall scores for each individual case, as well as combined cases, are illustrated in Table 3. These results reflect the level of agreement amongst the three markers in relation to students’ overall scores, out of 24. As can be seen in Table 3, Case 3 achieved the highest ICC of 0.80 (CI: 0.56–0.93), while Case 4, had the lowest ICC of 0.09 (CI: −1.03–0.86). When all cases were combined, the ICC demonstrated good reliability (0.70; CI 0.58–0.80).

Overall, based on tutor, consumer, and self-marked (students) rubrics, 20.8%, 14.6% and 10.4% of student participants failed the assessment, respectively (*n* = 96). A significant difference was found between tutors and students (*p* = 0.006), but not between consumers and students (*p* = 0.29) or tutors and consumers (*p* = 0.15), in relation to pass/fail categories, using McNemar’s Test.

Table 4 illustrates the Fleiss’ Kappa statistic for each individual case, as well as combined cases, across the three markers. Similar to ICC, the value (−0.06–0.82) and significance of the Fleiss’ Kappa statistic varied across cases, but was significant for combined cases (0.57, *p* < 0.001).

Test re-test reliability analyses of 58 role-plays audio-recorded in 2017 and 2018 indicated high agreement across all four cases (2017–2018), as can be seen in Table 5. Analyses conducted on combined cases resulted in a high correlation (0.87; *p* < 0.001), indicating the rubric was psychometrically sound, in terms of its test re-test reliability.

## 4. Discussion

This study explores the reliability of a rubric developed and used in the first and only, to our knowledge, three studies in the literature which employed observed behavioral measurement post-MHFA training [20,21,29]. Due to the potential for the discrepancy between self-reported and observed behaviors [21], it is important to assess participants’ ability to “show how” they apply their newly acquired knowledge and skills post-training [35]. ICC demonstrated poor-to-good reliability for four cases, poor-to-excellent reliability for one case, and moderate-to-excellent reliability for one case, as well as for combined cases overall [36]. Fleiss’ Kappa also varied across cases but was shown to be fair-to-good for combined cases [34]. Test re-test reliability was high for each individual case and combined cases, as indicated by r values ranging from 0.77–0.96 [37]. Overall, when cases were combined, the rubric demonstrated good reliability; however, there was variability between cases highlighting the need for further improvements to ensure clarity and consistency. Due to the variations in IRR, modifications to the rubric are recommended, such as the inclusion of clear examples under each item stem, developing a scoring guide, indicating which items must be performed to pass the assessment, and creating two versions of the rubric for suicidal and non-suicidal cases.

Despite sound psychometric properties for combined cases, the IRR analyses indicated that the reliability of the rubric varies across cases and items. As can be seen in Table 4, Cases 5 and 6 demonstrated negative Kappa values, indicating that ‘observed agreement is less than that expected from chance alone’ [34]. Case 2 demonstrated a Kappa value indicating poor agreement, while fair-to-good agreement was demonstrated for Cases 3 and 4, as well as for combined cases [34]. Only Case 1 had a Kappa value demonstrating an excellent level of agreement [34]. For the ICC analyses, the values demonstrated poor-to-good reliability for Cases 1, 4, 5, and 6, poor-to-excellent for Case 2, and moderate-to-excellent for Case 3, as well as for combined cases [36]. It is also evident from Table 2 that the percentage agreement greatly varies depending on the item (39.93–94.45%). These variations may be attributed to a lack of examples as to how each item may be performed by the participant. Rubrics can be a reliable tool to measure performance when they are accompanied by examples [38]. As can be seen in Appendix B and Appendix C, which illustrate two modified versions of the rubric, examples are recommended under all 10 items of the rubric to ensure clarity among assessors for each item. The item with the lowest percentage agreement, “Appropriate Follow-up Actions” (39.93%), was removed from both rubrics, and the required follow-up actions were incorporated as examples within the item “Takes appropriate action” in the modified rubrics, where relevant. Other modifications were incorporated to ensure the rubric reflected the current MHFA Australia guidance. For example, item 3 was changed to “*Listens and communicates* non-judgmentally” to align with ALGEE in the fourth edition of the MHFA manual [9].

Prior research on the development of rubrics to assess pharmacy students’ asthma first aid skills post-training has indicated the importance of including and highlighting items that students must perform to pass a case [17]. However, due to the nature of Asthma First Aid simulation exercises, there is debate regarding the minimum standards a student is required to perform during simulated assessment [17]. Unlike Asthma First Aid whereby the signs of an acute exacerbation of asthma symptoms may be immediately apparent, the signs of a mental health crisis, such as suicidal thoughts and behaviors, may not be immediately apparent, and the MHFAider often needs to ascertain whether the person is experiencing a mental health crisis by asking appropriate questions, such as asking about suicidal thoughts and behaviors directly [9]. Therefore, if a person displays potential suicide warning signs, such as the verbal cues, social or medical history, and physical signs or symptoms described in the suicidal cases in Appendix A, then an MHFAider should assess for suicide by asking directly, as per the MHFA manual [9]. Furthermore, an MHFAider needs to demonstrate that they have picked up these cues, and accordingly assessed for suicide by asking directly [9]. Therefore, item 5 (Table 1) must be performed, for the student to pass if the simulated patient displays signs, symptoms, and cues indicating that they may be experiencing thoughts of suicide, as is the case for all the scenarios included in this study (Appendix A). It is evident from Table 2, that there was a high percentage agreement (94.45 %) for item 5 regarding assessing for suicide, in that it is clear to assessors whether the MHFAider has done this or not. However, the appropriate action to take, afterward, indicated in item 9 is not as clear to assessors, as indicated by a lower percentage agreement of 61.46%. This may be because the appropriate action is dependent on whether the person responds that they are having suicidal thoughts or not. If the person expresses current suicidal thoughts, then a MHFAider is required to keep them safe, by not leaving them alone (e.g., staying with them, calling family member/friend/partner), and connecting them to immediate professional help (e.g., doctor, mental health crisis team or ambulance) [9]. If a student does not perform this action, then they have not cared for the suicidal simulated patient appropriately. However, if the simulated patient responds that they are not having current suicidal thoughts, while it is still important to provide MHFA and follow ALGEE, it is not necessary to stay with the person and ensure they receive immediate professional help, as they are not experiencing a mental health crisis [9]. The original rubric (Table 1) did not differentiate between these two scenarios. The modified rubrics (Appendix B and Appendix C) differentiate between these two scenarios and clearly indicate the barrier items that must be performed to pass the case.

Furthermore, the analyses also indicated that students may not have always been aware of what constituted a pass or fail for the assessments, as they were significantly less like to score themselves in a manner indicating that they had failed the case, when compared to tutors. Moreover, the consumer was found to provide significantly higher scores across all cases, in comparison to the tutor and student, indicating that they may have been less familiar with what constitutes appropriate MHFA actions. While it may have been clear to the tutor, who is often an accredited MHFA instructor, which items must be performed to ensure the simulated patient’s health and safety, and ultimately pass the case, it may be less clear to consumers and students when marking. As can be seen in Appendix B and Appendix C, it is recommended to highlight these barrier items by shading and providing clear instruction, such as ‘*Participant MUST assess for suicide risk to pass the case*’. Furthermore, to ensure clarity and simplicity of the rubric, it is also recommended that the examples under each item are adapted depending on the nature of the case. Hence, two different versions of the rubric were developed to be used for suicidal and non-suicidal cases (Appendix B and Appendix C). Both rubrics contain 10 items; however, the rubric in Appendix B contains an item (6) relating to appropriate follow-up questions for a person experiencing thoughts of suicide, but does not contain an item relating to encouraging self-help as this is less relevant for a person experiencing a crisis, such as suicide. The rubric in Appendix C, which is to be used when a person responds that they are not experiencing thoughts of suicide, does not contain an item relating to asking appropriate follow-up questions about suicide as this is not relevant, but does contain an item relating to encouraging self-help. All other items in both rubrics are similar and contain examples that are relevant to providing MHFA to a person who is (Appendix B) and is not (Appendix C) experiencing thoughts of suicide.

The addition of scoring guides may improve the clarity of the rubric, which may, in turn, highlight expectations to educators and students and reduce ambiguity among diverse participant populations [39]. Furthermore, the scoring guides provide instructions to assessors, regarding the meaning of different colors, fonts, and superscripts within the rubric. In addition to its main function in assessing performance, the rubric can also be used to stimulate conversation around key points during the on-the-spot performance feedback discussion [39]. Feedback that is individualized, collaborative, and supportive of self-awareness “works best” in medical education, and immediate feedback is recommended for difficult tasks [19]. Providing MHFA to a person with lived experience in mental illness during role-play assessments has been described as a “challenging” and “scary” assessment, although “rewarding” [29], indicating that immediate feedback is an important and necessary part of the assessments.

The simulated patient role-plays have the potential to benefit participating and observing students, due to their relevance to future practice as frontline healthcare professionals and by providing students with opportunities to practice important MHFA skills, as demonstrated by a qualitative evaluation of these assessments [29]. When students perceive their assessments to be relevant to their future practice, the assessments have the potential to motivate students to learn [19]. This is further supported by the fact that students who participate in and observe the simulated patient role-plays are more likely to have sustained improvements in confidence post-training in comparison to students who completed MHFA training, but did not participate in or observe the role-plays [7]. Given that MHFA training is often delivered to healthcare students, including medicine and nursing students [8], this rubric has the potential for widespread use among MHFA participants and in healthcare curricula to promote learning and to assess participants on their ability to provide MHFA. By ensuring that the rubric is reliable across assessors and over time, we can ensure that assessments are standardized and facilitate comparisons across study sites and populations.

### Strengths and Limitations

This study describes the reliability testing of the only rubric designed to assess actual, observed behaviors post-MHFA training during simulated patient role-plays. There is evidence to support the effectiveness of MHFA in improving mental health knowledge and literacy, as well as self-reported helping behaviors, recognition of mental illness, and confidence and intentions relating to MHFA provision [10,11]. This study is among the first to explore actual, observed behaviors post-training and starts to fill the gaps in the literature by exploring methods to facilitate the assessment of how MHFA skills are actually used by participants post-training. Nonetheless, despite these strengths, certain limitations require that the findings of this study be interpreted with caution. The demographic characteristics of participating students were not captured during data collection, as the study involved education and training that is integrated into the pharmacy curriculum. To minimize disruptions to students’ learning, data were collected during routine classroom activities, and no further information was requested from students. Hence, future research exploring differences in students’ skills based on demographic characteristics may be warranted. It is also important to note that multiple consumers participated in this study and may have not strictly followed the case script, at times, or had to improvise, due to questions by the student which were not anticipated. In general, the cases described in Appendix A were performed consistently; however, even minor individual variations may have impacted assessors’ marking of the rubric. Another potential limitation that may have affected the IRR of the rubric was the tutor’s knowledge and familiarity with professional standards and MHFA course content in comparison to students and consumers, as he/she was an MHFA instructor and/or pharmacist who had completed MHFA training and may have had certain expectations, due to his/her familiarity with the profession and MHFA training, which may not be as well-known to students and consumers. However, through the recommendations made in the Discussion, it is anticipated that these differences will be minimized by adding a scoring guide, pass/fail barrier items, and item-specific examples. Given that the rubrics in Appendix B and Appendix C are modified, they also warrant further psychometric testing to ensure reliability. Furthermore, given that some cases involved in this study were role-played by limited sample size (e.g., Case 4), the IRR findings for these cases may not be accurate. It should also be noted that the Pearson’s correlation for Case 4 was not significant. Hence, it may be beneficial to conduct further reliability testing on a larger sample size using the modified rubrics. Regarding the test re-test reliability analyses, a limitation of this study is that these analyses were conducted using the audio-recordings. The study could have been strengthened by conducting these analyses using video-recordings, which would have enabled the assessment of non-verbal communication skills. Whilst video-recording should be considered in future research, the researchers should take into consideration that consumers and students may have lower acceptance of this data collection method and attempt to provide flexibility with respect to the recording. It is important to note that a strength of the study lies in the fact that an independent rater marked the audio recordings at two time points for the test re-test reliability analyses. Finally, it is important to recognize that not all people experiencing mental health problems and crises require suicide assessment, as was the case for the scenarios used in this study. Future scenarios may not require the MHFAider to assess for suicide, as the appropriate actions to be taken when caring for someone experiencing other mental health problems or crises (e.g., mania or a panic attack) differ and may not always involve assessment of suicidal thoughts, as required in this study for the six depression and anxiety scenarios developed for this purpose (Appendix A). The modified rubrics in this study are recommended for use in scenarios where suicide assessment is required by the MHFAider, and future research exploring the development of assessments and rubrics for SP cases relating to other mental health problems (e.g. substance use) and crises (e.g. panic attacks) is warranted. Furthermore, further research exploring their reliability across different settings and populations is also needed to allow for comparisons with the current findings.

## 5. Conclusions

MHFA training is available internationally and has been shown to improve self-reported knowledge, attitudes, and behaviors in relation to people living with mental illness. Research focusing on post-training observed behavioral measurement is limited. A rubric was developed to assess participants’ observed MHFA skills during simulated patient role-plays, thereby contributing to the evidence base surrounding observed behavioral measurement post-MHFA training. This study has reported on the psychometric testing of this rubric and found that while its test re-test reliability is relatively stable, its IRR varies across cases. This has led to evidence-based recommendations to improve clarity and the reliability of the rubric across assessors. Future studies exploring the reliability and validity of the modified rubrics, across diverse participant populations, are warranted.

## Figures and Tables

**Table 1:** Sample rubric (prior to reliability analyses).

	Action	Yes (2)	Partial (1)	NO (0)	N/A
**1**	**Approaches the patient appropriately**				
**2**	**Provides a comfortable setting for the patient to talk**				
**3**	**Listens non-judgmentally**				
**4**	**Asks appropriate open-ended questions** (e.g., how long have you been feeling this way? Have you sought any professional help? How are you coping?)				
**5**	**Asks if the patient is having suicidal thoughts**				
**6**	**Asks appropriate follow-up questions** (e.g., do they have a plan? Have they attempted before? Are they taking alcohol/drugs?)				
**7**	**Gives reassurance and appropriate information** (e.g., Tells patient they care and want to help, state thoughts of suicide are often associated with treatable mental illness, tells person thoughts of suicide are common and do not have to be acted on)				
**8**	**Displays empathy**				
**9**	**Takes appropriate action** (does not leave the patient alone, connects with professional help, such as Lifeline or the Suicide Call Back Service, connect with a family member to pick up or immediately see the GP)				
**10**	**Encourages self-help** (e.g., looking after self, support groups for those who lost a partner)				
**11**	**Good non-verbal communication**				
**12**	**Appropriate follow-up actions** (e.g., takes phone number to call for follow-up, continual willingness to help)				

**Table 2:** Percentage agreement for each item among three markers.

Item Number	Overall Percentage Agreement
1 (Approach)	74.30%
2 (Setting)	60.77%
3 (Listen)	75.70%
4 (Ask)	57.99%
5 (Suicide assessment)	94.45%
6 (Suicide follow-up)	64.93%
7 (Reassurance)	50.39%
8 (Empathy)	56.59%
9 (Action)	61.46%
10 (Self-help)	52.08%
11 (Non-verbal)	61.81%
12 (Follow-up)	39.93%

**Table 3:** ICC values for overall scores, per case.

	Total Case N	ICC Average	95% Confidence Interval
Case 1	20	0.74	0.46–0.89
Case 2	18	0.75	0.45–0.90
Case 3	16	0.80	0.56–0.93
Case 4	5	0.09	−1.03–0.86
Case 5	19	0.57	0.13–0.82
Case 6	18	0.40	−0.24–0.75
Combined cases	96	0.70	0.58–0.80

**Table 4:** Fleiss’ Kappa values for overall pass/fail for each case.

	Total Case N	Fleiss’ Kappa	*p* Value
**Case 1**	20	0.82	<0.001
**Case 2**	18	0.38	0.005
**Case 3**	16	0.73	0.000
**Case 4**	5	0.66	0.011
**Case 5**	19	−0.06	0.675
**Case 6**	18	−0.04	0.777
**Overall**	96	0.57	<0.001

**Table 5:** Test re-test reliability analyses based on audio-recording of cases 3–6.

	Total Case N	Mean	SD	Pearson Correlation	*p* Value
Case 3 test	16	18.69	3.071	0.96	<0.001
Case 3 re-test	16	17.88	2.941		
Case 4 test	5	18.00	1.414	0.82	0.093
Case 4 re-test	5	16.80	2.168		
Case 5 test	19	17.68	3.019	0.87	0.000
Case 5 re-test	19	17.53	2.547		
Case 6 test	18	17.94	1.893	0.77	0.000
Case 6 re-test	18	17.94	1.765		
Combined cases test	58	18.07	2.595	0.87	0.000
Combined cases re-test	58	17.69	2.386		

## Data Availability

The data are not publicly available due to privacy and ethical concerns.

## Appendix A

**Table A1:** Simulated patient cases scenarios.

Case Number (Year)	Case 1 (2016)	Case 2 (2016)	Case 3 (2017)	Case 4 (2017)	Case 5 (2018)	Case 6 (2018)
**Topic**	Depressive symptoms without suicidal thoughts.	Depressive symptoms with suicidal thoughts.	Depressive symptoms with suicidal thoughts.	Anxiety/depressive symptoms without suicidal thoughts.	Depressive symptoms with suicidal thoughts.	Anxiety/depressive symptoms with suicidal thoughts.
**Reason for pharmacy visit**	Feeling depressed for the past two months.	Request to fill antidepressant repeat.	Request multivitamin for energy.	Request sleeping tablet for insomnia.	Request multivitamin for energy.	Request to fill antidepressant repeat.
**Symptoms**	Teary and stressed. Severe sleep deprivation (first baby, three months old, unable to sleep for more than half an hour).	Deteriorating mood for the past two months after taking antidepressant (fluoxetine). Stopped taking fluoxetine a few days ago. Poor appetite. Little motivation for daily activities.	Flat and lethargic since recent divorce. Unable to get out of bed in the morning. Little motivation to work.	Insomnia.	Flat and lethargic. Low energy to get out of bed in the morning. Little motivation to go to work.	Feeling worse over the past few weeks and questioning whether life is worth it. Low motivation for self-care. Poor appetite.
**Medical history**	Previous depression diagnosis at 21. History of antidepressant use & psychotherapy.	Recent diagnosis of depression. Doctor prescribed fluoxetine last month to help with the mood.	No significant medical history.	Had panic attacks as a teenager.	History of depression in early 20s. Went through psychological therapy for months.	Citalopram for anxiety disorder for years.
**Social history**	New immigrant from America. Social isolation. Little support from partner (at work).	Recent financial struggles due to job cuts three months ago. Social isolation (partner at work; two daughters have moved out).	Divorced three months ago.Close to sister.	Has two young children (12 months and 22 months). Supportive parents and partner (working two jobs).	Recent divorce. Closer to sister.	Recent financial issues (house). Recent loss of employment (isolation). Supportive partner but rarely home (work).
**Suicidal thoughts**	No.	Current suicidal thoughts with no current plan. No previous attempt.	Current suicidal thoughts with no current plan. No previous attempt.	No.	Current suicidal thoughts with plan (considers jumping off the balcony). No previous attempt.	Current suicidal thoughts with plan (taking all citalopram at once).
**Alcohol/drug use**	½ bottle of red wine each night.	Taking sleeping tablets (doxylamine) every night. A whole bottle of wine on some nights.	Recent increase of alcohol intake	1–2 glasses of wine each night.	Recent increase of alcohol intake (one bottle of wine per night).	Taking sleeping tablets (doxylamine) every night. Recent increase in alcohol intake (2–3 of partner’s beers every day).
**Says**	“I can’t do this anymore”, “My baby would be better off without me.”	“Everyone would be better off without me. I don’t see a point in living anymore.”	“I just feel like it’s not worth it anymore”, “There’s no point in life.”	“It’s just too hard”, “I don’t know if I can keep doing this.”	“I just feel like it’s not worth it anymore”, “There’s no point in life.”	“Everyone would be better off without me. I don’t see a point in living anymore.”

## Appendix B

**Scoring Guide:**

- Actions shaded in GREY must be performed by the participant to pass the case.
- The participant must receive a score of at least 10/20 **AND** perform all actions shaded in GREY to pass the case.
- *Phrases and actions* that are *italicized* are merely suggestions and examples, and do not need to be articulated by the participant word-for-word.
- Phrases and actions that are underlined must be performed by the participant, to pass the item.

**Table A2:** Modified rubric for suicidal case.

Item	Action	Full Marks (2)	Partial Marks (1)	NO Marks (0)
**1**	**Approaches the consumer appropriately** *Introduces self* *Greets consumer*			
**2**	**Provides a comfortable setting for the consumer to talk** *Negotiates privacy* *Ensures the consumer is comfortable*			
**3**	**Listens and communicates non-judgmentally** *Does not blame the consumer* *Does not use words like “alcoholic”, “druggie”* *Non-judgmental attitudes displayed*			
**4**	**Asks appropriate open-ended questions** *How can I help?* *How long have you been feeling this way?* *What type of support have you sought?* *How are you coping?* *Who have you spoken to about your emotions?*			
**5**	**Asks if the consumer is having suicidal thoughts** • Asks directly *(e.g., Are you thinking of killing yourself/ending your life/suicide?).* If the participant asks using indirect language, only, (e.g., harming/hurting yourself), then only give partial marks (1). * Participant MUST assess for suicide risk to pass the case.			
**6**	**Asks appropriate follow-up questions in relation to consumer’s suicidal thoughts** *Do they have a plan? (e.g., how/when they will suicide)* Have they attempted suicide before? Are they using alcohol/drugs? Note: participants MUST enquire about a “plan”, otherwise “0” marks for this item. If the participant asks about the plan, only, give one partial mark. If the participant asks about the plan, and at least one other question, give the full two marks.			
**7**	**Gives reassurance and appropriate information** *They care and want to help* *Thoughts of suicide are often associated with treatable mental illness* *Thoughts of suicide are common and do not have to be acted on* *Mental illness is common* *Effective psychological/pharmacological treatment for mental illness exists*			
**8**	**Displays empathy** *Uses empathetic language (e.g., “I can see that this is a difficult time for you”)* *Does not use sympathetic language (e.g., “I feel sorry for you”)* *Demonstrates continual willingness to help*			
**9**	**Takes appropriate action** Does not leave the consumer alone (*e.g., connect with family member/friend to pick up, calls Mental Health Crisis Team/ambulance to pick up*) Connects the consumer with appropriate professional help (*e.g., Calling Mental Health Crisis Team/Ambulance, Lifeline/Suicide call back service or immediately see the doctor*) * Participant MUST do both of these actions to pass the case and receive full marks (2) for this item. Additional examples of follow-up actions Takes phone number to call for follow-up Provides pharmacy number for consumer to call back			
**10**	**Good non-verbal communication** *Open body language* *Appropriate eye contact* *Non-judgmental facial expressions*			
**TOTAL (out of 20)**				

## Appendix C

**Scoring Guide:**

- Actions shaded in GREY must be performed by the participant to pass the case.
- The participant must receive a score of at least 10/20 AND perform all actions shaded in GREY to pass the case.
- *Phrases and actions* that are *italicized* are merely suggestions and examples, and do not need to be articulated by the participant word-for-word.

**Table A3:** Modified rubric for non-suicidal case.

Item	Action	Full Marks (2)	Partial Marks (1)	NO Marks (0)
**1**	**Approaches the consumer appropriately** *Introduces self* *Greets consumer*			
**2**	**Provides a comfortable setting for the consumer to talk** *Negotiates privacy* *Ensures the consumer is comfortable*			
**3**	**Listens and communicates non-judgmentally** *Does not blame the consumer* *Does not use words like “alcoholic”, “druggie”* *Non-judgmental attitudes displayed*			
**4**	**Asks appropriate open-ended questions** *E.g., “How can I help?”* *E.g., “How long have you been feeling this way?”* *E.g., “What type of support have you sought?”* *E.g., “How are you coping?”* *E.g., “Who have you spoken to about your emotions?”*			
**5**	**Asks if the consumer is having suicidal thoughts** Asks directly (*e.g., “Are you thinking of killing yourself/ending your life/suicide?”*). If the participant asks using indirect language, only, (*e.g., harming/hurting yourself*), then only give partial marks (1). * Participant MUST assess for suicide risk to pass the case.			
**6**	**Gives reassurance and appropriate information** *They care and want to help* *Mental illness is common* *Effective psychological/pharmacological treatment for mental illness exists*			
**7**	**Displays empathy** *Uses empathetic language (e.g., “I can see that this is a difficult time for you”)* *Does not use sympathetic language (e.g., “I feel sorry for you”)* *Demonstrates continual willingness to help*			
**8**	**Takes appropriate action** *Offers a range of professional services (e.g., GP, Beyondblue, Black Dog Institute, psychologist, psychiatrist)* *Encourages discussing with family member/friend/partner* *Takes phone number to call for follow-up* *Provides pharmacy number for consumer to call back*			
**9**	**Encourages self-help** *Lifestyle recommendations (e.g., exercise, eating well)* *Relevant support groups (e.g., widowers, carers, divorcees, new mothers)*			
**10**	**Good non-verbal communication** *Open body language* *Appropriate eye contact* *Non-judgmental facial expressions*			
**TOTAL (out of 20)**				

## References

[1] Rehm J., Shield K.D. (2019). Global Burden of Disease and the Impact of Mental and Addictive Disorders. Curr. Psychiatry Rep..

[2] World Health Organisation Suicide Data. https://www.who.int/mental_health/prevention/suicide/suicideprevent/en/.

[3] World Health Organisation World Health Report: Mental Disorders Affect One in Four People. https://www.who.int/whr/2001/media_centre/press_release/en/.

[4] Australian Bureau of Statistics (2007). National Survey of Mental Health and Well-Being: Summary of Results.

[5] International Pharmaceutical Federation (FIP) (2015). Focus on Mental Health: The Contribution of the Pharmacist.

[6] Pharmaceutical Society of Australia (2013). Mental Health Care Project: A Framework for Pharmacists as Partners in Mental Health Care.

[7] Boukouvalas E., El-Den S., Murphy A.L., Salvador-Carulla L., O’Reilly C.L. (2019). Exploring Health Care Professionals’ Knowledge of, Attitudes Towards, and Confidence in Caring for People at Risk of Suicide: A Systematic Review. Arch. Suicide Res..

[8] El-Den S., Moles R., Choong H.J., O’Reilly C. (2020). Mental Health First Aid training and assessment among university students: A systematic review. J. Am. Pharm. Assoc..

[9] Kitchener B.A., Jorm A.F., Kelly C.M. (2017). Mental Health First Aid Manual.

[10] Morgan A.J., Ross A., Reavley N.J. (2018). Systematic review and meta-analysis of mental health first aid training: Effects on knowledge, stigma, and helping behaviour. PLoS ONE.

[11] Hadlaczky G., Hökby S., Mkrtchian A., Carli V., Wasserman D. (2014). Mental Health First Aid is an effective public health intervention for improving knowledge, attitudes, and behaviour: A meta-analysis. Int. Rev. Psychiatry.

[12] O’Reilly C., Bell J., Kelly P., Chen T. (2011). Impact of mental health first aid training on pharmacy students’ knowledge, attitudes and self-reported behaviour: A controlled trial. Aust. N. Z. J. Psychiatry.

[13] Lipson S.K., Speer N., Brunwasser S., Hahn E., Eisenberg D. (2014). Gatekeeper training and access to mental health care at universities and colleges. J. Adolesc. Health.

[14] Reavley N.J., Morgan A.J., Fischer J.A., Kitchener B., Bovopoulos N., Jorm A.F. (2018). Effectiveness of eLearning and blended modes of delivery of Mental Health First Aid training in the workplace: Randomised controlled trial. BMC Psychiatry.

[15] Seyed Nematian S.S., Palenik C.J., Mirmasoudi S.K., Hatam N., Askarian M. (2017). Comparing knowledge and self-reported hand hygiene practices with direct observation among Iranian hospital nurses. Am. J. Infect. Control.

[16] Prince S.A., Adamo K.B., Hamel M.E., Hardt J., Gorber S.C., Tremblay M. (2008). A comparison of direct versus self-report measures for assessing physical activity in adults: A systematic review. Int. J. Behav. Nutr. Phys. Act..

[17] Luckie K., Saini B., Galstaun V., Kritikos V., Collins J.C., Moles R.J. (2018). The effectiveness of an online training programme to prepare teachers to provide asthma first aid. J. Paediatr. Child Health.

[18] Girard J., Cohn J. (2016). A Primer on Observational Measurement. Assessment.

[19] Scott I.M. (2020). Beyond ‘driving’: The relationship between assessment, performance and learning. Med. Educ..

[20] Boukouvalas E., El-Den S., Chen T., Moles R., Saini B., Bell A., O’Reilly C. (2018). Confidence and attitudes of pharmacy students towards suicidal crises: Patient simulation using people with a lived experience. Soc. Psychiatry Psychiatr. Epidemiol..

[21] El-Den S., Chen T.F., Moles R.J., O'Reilly C. (2018). Assessing Mental Health First Aid Skills Using Simulated Patients. Am. J. Pharm. Educ..

[22] Rollnick S., Butler C.C., Kinnersley P., Gregory J., Mash B. (2010). Motivational interviewing. BMJ.

[23] Xu T., de Almeida Neto A.C., Moles R.J. (2012). Simulated caregivers: Their feasibility in educating pharmacy staff to manage children’s ailments. Int. J. Clin. Pharm..

[24] Miller W.R. (1983). Motivational Interviewing with Problem Drinkers. Behav. Psychother..

[25] Stellmack M.A., Konheim-Kalkstein Y.L., Manor J.E., Massey A.R., Schmitz J.A.P. (2009). An Assessment of Reliability and Validity of a Rubric for Grading APA-Style Introductions. Teach. Psychol..

[26] Moskal B.M., Leydens J.A. (2000). Scoring rubric development: Validity and reliability. Pract. Assess. Res. Eval..

[27] Brown M.C., Conway J., Sorensen T.D. (2006). Development and Implementation of a Scoring Rubric for Aseptic Technique. Am. J. Pharm. Educ..

[28] Rossetto A., Jorm A., Reavley N. (2014). Examining predictors of help giving toward people with a mental illness: Results from a national survey of Australian adults. SAGE Open.

[29] O’Reilly C., Moles R., Boukouvalas E., El-Den S. (2019). Assessing students’ mental health crisis skills via consumers with lived experience: A qualitative evaluation. J. Ment. Health Train. Educ. Pract..

[30] One Door Mental Health. https://www.onedoor.org.au/.

[31] American Psychiatric Association (2013). Diagnostic and Statistical Manual of Mental Disorders: DSM-V.

[32] IBM Corp (2016). IBM SPSS Statistics for Windows, Version 24.0.

[33] Collins J.C., Chan M.Y., Schneider C.R., Yan L.R., Moles R.J. (2020). Measurement of the reliability of pharmacy staff and simulated patient reports of non-prescription medicine requests in community pharmacies. Res. Soc. Adm. Pharm..

[34] Gisev N., Bell J.S., Chen T.F. (2013). Interrater agreement and interrater reliability: Key concepts, approaches, and applications. Res. Soc. Adm. Pharm..

[35] Miller G.E. (1990). The assessment of clinical skills/competence/performance. Acad. Med..

[36] Koo T.K., Li M.Y. (2016). A Guideline of Selecting and Reporting Intraclass Correlation Coefficients for Reliability Research. J. Chiropr. Med..

[37] Karras D.J. (1997). Statistical methodology: II. Reliability and variability assessment in study design, Part A. Acad. Emerg. Med. Off. J. Soc. Acad. Emerg. Med..

[38] Jonsson A., Svingby G. (2007). The use of scoring rubrics: Reliability, validity and educational consequences. Educ. Res. Rev..

[39] Lasater K. (2007). Clinical Judgment Development: Using Simulation to Create an Assessment Rubric. J. Nurs. Educ..

